# Supplementary material for: Comparative genomics provides new insights into the diversity, physiology, and sexuality of the only industrially exploited tremellomycete: Phaffia rhodozyma
Source: BMC Genomics. 2016 Nov 9;17:901. doi: 10.1186/s12864-016-3244-7 (PMC5103461; doi:10.1186/s12864-016-3244-7)
Supplement: Additional file 6: — List of orphan genes with links to PFAM (related to Additional file 1: Table S1). (ZIP 1428 kb) [file 12864_2016_3244_MOESM6_ESM.zip › PFAM_UNIQUE/G03552_P.html]

#                                                                            --- full sequence --- -------------- this domain -------------   hmm coord   ali coord   env coord
  
# target name        accession   tlen query name           accession   qlen   E-value  score  bias   #  of  c-Evalue  i-Evalue  score  bias  from    to  from    to  from    to  acc description of target
  
#------------------- ---------- ----- -------------------- ---------- ----- --------- ------ ----- --- --- --------- --------- ------ ----- ----- ----- ----- ----- ----- ----- ---- ---------------------
  
G03552\_P             -            671 Dicty\_REP            PF05086.7    911       1.5    5.1   4.9   1   1    0.0035       2.1    4.6   4.9   250   278   166   194   139   251 0.53 - Dictyostelium (Slime Mold) REP protein  
G03552\_P             -            671 Ecl1                 PF12855.2     43   5.5e-06   24.3   0.4   1   2   0.00035      0.69    7.9   0.0     8    19    15    29    13    45 0.75 - Life-span regulatory factor  
G03552\_P             -            671 Ecl1                 PF12855.2     43   5.5e-06   24.3   0.4   2   2   4.1e-06    0.0082   14.1   0.1    20    42   229   251   211   252 0.88 - Life-span regulatory factor  
G03552\_P             -            671 FliC                 PF12445.3    129       7.1    5.4   6.7   1   1    0.0091        18    4.1   6.7    33    65   156   188   134   202 0.65 - Flagellin protein
